# Gene regulation for inflammation and inflammation resolution differs between umbilical arterial and venous endothelial cells

**DOI:** 10.1038/s41598-023-43142-6

**Published:** 2023-09-27

**Authors:** Julia Caroline Michaeli, Sebastian Albers, Carolina de la Torre, Yannick Schreiner, Sara Faust, Thomas Michaeli, Daniel Tobias Michaeli, An Liying, Bernhard K. Krämer, Ksenija Stach, Benito A. Yard

**Affiliations:** 1 5th Medical Department, University Hospital Mannheim, Heidelberg University, Theodor-Kutzer-Ufer 1-3, 68167 Mannheim, Germany; 2 Department of Obstetrics and Gynecology, LMU University Hospital, LMU Munich, Munich, Germany; 3 Department of Orthopaedics and Sport Orthopaedics, School of Medicine, Klinikum Rechts Der Isar, Technical University of Munich, Munich, Germany; 4 Institute of Clinical Chemistry, Medical Faculty Mannheim, Heidelberg University, Heidelberg, Germany; 5 Division of Personalized Medical Oncology, German Cancer Research Center (DKFZ), Heidelberg, Germany; 6 DKFZ-Hector Cancer Institute, the University Medical Center Mannheim, Mannheim, Germany; 7 Department of Personalized Oncology, University Hospital Mannheim, Heidelberg University, Mannheim, Germany; 8 European Center for Angioscience, Mannheim, Germany

**Keywords:** Acute inflammation, Mechanisms of disease

## Abstract

Systemic inflammation affects the whole vasculature, yet whether arterial and venous endothelial cells differ in their abilities to mediate inflammation and to return to homeostasis after an inflammatory stimulus has not been addressed thoroughly. We assessed gene-expression profiles in isolated endothelial cells from human umbilical arteries (HUAEC) or veins (HUVEC) under basal conditions, after TNF-α stimulation and various time points after TNF-α removal to allow reinstatement of homeostasis. TNF-α regulates the expression of different sets of transcripts that are significantly changed only in HUAEC, only in HUVEC or changed in both. We identified three types of gene regulation, i.e. genes that were significantly regulated after 24 h of TNF-α stimulation but no longer when TNF-α was removed (homeostatic regulation), genes that maintained significantly regulated after TNF-α removal (not homeostatic regulation) and genes that were only significantly regulated when TNF-α was removed (post-regulation). HUAEC and HUVEC quantitatively differed in these types of gene regulation, with relatively more genes being post-regulated in HUAEC. In conclusion our data demonstrate that HUAEC and HUVEC respond intrinsically different to an inflammatory insult. Whether this holds true for all endothelial cells and its relevance for inflammatory insults in different organs during systemic inflammation warrants further studies.

## Introduction

Structural heterogeneity of endothelial cells (EC) is widely acknowledged and is a consequence of differential regulation of EC structure and function within blood vessels of different tissues^1^. Differences in gene expression profiles are at the heart of EC heterogeneity and dictate a broad spectrum of phenotypic differences that match the need of the endothelium in their distinct local environment. Therefore, not only macro and micro vascular EC may differ within one organ^2–4^, but also EC that are embedded in venous or arterial blood vessels likely differ due to the different functions of arteries and veins^5^.

Vascular EC are major targets of a myriad of inflammatory mediators and cells/components of the innate immune system. In response to pro-inflammatory cytokines such as TNF-α and IL-1, EC express adhesion molecules on their surface which permits leucocyte rolling, firm adhesion and egress from the circulation^6,7^. In acute systemic inflammation, e.g. sepsis or septic shock, the barrier function of the endothelium is severely compromised. This further propagates the inflammatory response and contributes to adverse outcomes in these patients.

Inflammation is inevitably associated with collateral tissue damage due to the production of reactive oxygen species (ROS) and many cytokines that are released in the inflamed tissue. Active regulation of the inflammatory process is of utmost importance to limit collateral damage to the EC and to prevent severe tissue remodeling, fibrosis and loss of tissue functionality. Hence, inflammation resolution is of equal importance as inflammation since it aims to reinstate tissue integrity after an inflammatory insult. Impairment of inflammation resolution may underlie the cause of prevalent chronic inflammatory diseases, such as arthritis, atherosclerosis, and cardiovascular diseases^8,9^.

Inflammation resolution is classically defined as the period between the peak of inflammatory cells in the tissue and restoration of tissue homeostasis. It is widely perceived that inflammation resolution is subject to a tightly regulated network of processes^10^, starting with weaning off the synthesis of pro-inflammatory mediators and to inactivate those that are still around. Whether EC heterogeneity amongst different blood vessels within one organ also accounts for differences in resolution or the ease to return to homeostasis after inflammation is currently not known.

Although human umbilical vein endothelial cells (HUVECs), human coronary artery endothelial cells and human dermal microvascular endothelial cells differ in responsiveness to inflammatory mediators^11,12^, differences in global transcriptional regulation between arterial and venous EC under inflammatory conditions and under conditions that lead to return to homeostasis have not been well clarified. In keeping with EC heterogeneity and the notion that many inflammatory mediators harbor genetic polymorphisms that influence their expression, a direct comparison between genetically identical arterial and venous endothelial cells isolated from the same anatomical site would be preferable. To the best of our knowledge such studies have not been performed. Therefore, the current study sought to assess differences in transcriptional regulation between genetically identical arterial and venous endothelial cells that were cultured under basal conditions, that were exposed to TNF-α or that were exposed to TNF-α followed by medium change to allow return to homeostasis. We hypothesize that arterial and venous EC differ in their response to inflammation, their ability to mediate inflammation and to return to homeostasis after an inflammatory stimulus.

## Materials and methods

### Cell culture

Human umbilical vein endothelial cells (HUVEC) and Human umbilical arterial endothelial cells (HUAEC) were isolated from the same fresh human umbilical cords by inserting an IV cannula and a three-way valve on both ends of the umbilical artery and vein respectively. The blood vessels were thoroughly flushed with PBS before 5 ml of collagenase solution (1 mg/ml) (Merck, Darmstadt, Germany) was added. After clamping of the vessels, the umbilical cord was incubated for 10 min at 37 °C. Hereafter, the artery and vein were flushed with 15 ml of PBS to collect the endothelial cells. The cells were cultured in 1% gelatin (Fluka, Neu-Ulm, Germany) coated flasks in endothelial cell growth medium (PromoCell, Heidelberg, Germany) supplemented with essential growth factors and 5% fetal bovine serum (Gibco, Carlsbad, USA). Cultures were maintained at 37 °C, 95% relative humidity and 5% CO2. Confluent monolayers were passaged by TrypLE™ Select Enzyme (Thermo Fisher Scientific, Braunschweig, Germany) and experiments were conducted on cells in passage 2 or 3 at approximately 90–100% confluence. Umbilical cords were obtained from healthy women (Department of Obstetrics, University Medical Center Mannheim) after written informed consent. Isolation was approved by the local ethics committee (Medizinische Ethikkommission II der Medizinischen Fakultät Mannheim Ruprechts-Karls-University Heidelberg (Approval number: 2015-518-MA)). All research was performed in accordance with the Declaration of Helsinki and in accordance with relevant guidelines/regulations.

### Experimental conditions

For each umbilical cord donor, four T25 flasks of HUVECs and HUAECs were stimulated for 24 h with 25 ng/ml TNF-α (PeproTech, Hamburg, Germany). Hereafter, RNA was either directly isolated or alternatively after additional 12 or 24 h of culturing in the absence of TNF-α. RNA isolated from cells that were cultured in normal culture medium for 24 h was included for each donor to assess basal gene expression.

### RNA Isolation and microarray analysis

RNA isolation and microarray analysis were in essence performed as previously described^13^. In brief, total RNA was prepared using Trizol reagent (Ambion, Carlsbad, USA). RNA quality was confirmed by capillary electrophoresis on an Agilent 2100 bioanalyzer (Agilent Technologies, Palo Alto, CA). All RNA preparations were subjected to treatment with RNase free DNase I (Ambion, Carlsbad, USA) according to the manufacturer’s instructions. Gene expression profiling was performed using HuGene-2_0-st-type arrays (Affymetrix, Santa Clara, USA). Biotinylated antisense cDNA was prepared according to the Affymetrix standard labelling protocol with the GeneChip® WT Plus Reagent Kit and the GeneChip® Hybridization, Wash and Stain Kit (both from Affymetrix). The arrays were subsequently hybridized in a GeneChip Hybridization oven 640, stained in a GeneChip Fluidics Station 450 and scanned with a GeneChip Scanner 3000 (all from Affymetrix).

### Data processing and statistical analysis

A Custom CDF Version 25 with Entrez-based gene definitions was used to annotate the arrays^14^. Raw fluorescence intensity values were RMA background corrected and normalized, applying quantile normalization. Differential gene expression was analyzed based on loglinear mixed model ANOVA, using a commercial software package SAS JMP10 Genomics, version 15, from SAS (SAS Institute, Cary, NC, USA). A false positive rate of a = 0.05 with FDR correction was taken as the level of significance. Gene Set Enrichment Analysis (GSEA) was used to determine whether defined lists (or sets) of genes exhibit a statistically significant bias in their distribution within a ranked gene list using the fgsea package (Sergushichev, 2016) and ran under the open-source computer software R v3.4.0 (R Core Team, 2017). Pathways belonging to various cell functions such as cell cycle or apoptosis were obtained from public external databases (KEGG, http://www.genome.jp/kegg). For particular lists of differentially expressed genes (DEGs), GO and GEA were performed with clusterProfiler package, which supports statistical analysis and visualization of functional profiles for genes and gene clusters. The raw and normalized data are deposited in the Gene Expression Omnibus database (http://www.ncbi.nlm.nih.gov/geo/; accession No. GSE179478).

### Quantitative PCR

For confirmation of the Affymetrix data set, the expression level of selected genes was assessed by qPCR as previously described^13^. To this end, 1 µg of total RNA was reverse-transcribed into cDNA using the High-Capacity cDNA Reverse Transcription Kit (Applied Biosystems, Foster City, USA). RT reactions were carried out using the following protocol: 16 °C for 30 min, followed by 42 °C for 30 min and 85 °C for 5 min in a 2720 Thermal Cycler (Applied Biosystems). Samples were stored at − 20 °C until use. Quantitative PCR was performed on a Step-one Plus PCR System (Applied Biosystems) using TaqMan fast advanced master mix (Applied Biosystems) and the following TaqMan probes (Applied Biosystems): HEY2 (ID: Hs01012057_m1), DLL4 (ID: Hs00184092_m1), EFNB2 (ID: Hs00187950_m1), EPHB4 (ID: Hs00174752_m1), EMCN (ID: Hs01038204_m1), NR2F2 (ID: Hs00819630_m1), ICAM-1 (ID: Hs00164932_m1), VCAM-11 (ID: Hs 01003372_m1), SELE (ID: Hs00174057_m1), TXNIP (ID Hs00197750_m1), SLC4A4 (ID: Hs01047033_m1), PDE5A (ID: Hs00153649_m1), CXCL3 (ID: Hs00171061_m1), CXCL5 (ID: Hs00171085_m1) and ACTB (β-actin, ID: Hs 01060665_g1). The following thermal cycling profile was used for all samples: 20 s at 95 °C followed by 40 cycles of 1 s at 95 °C and 20 s at 60 °C.

For micro RNA expression, total RNA was reverse transcribed in 15 μL reactions, consisting of 7 μL RT Master Mix (1.5 μL 10 × RT Buffer, 0.15 μL 100 mM dNTPs, 1 μL MultiscribeTM Reverse Transcriptase (50U/μL), 0.19 μL RNase Inhibitor (20U/μL) and 4,16 μL nuclease-free water), 3 μL of specific RT Primers (hsa-miR-100 (000,437), hsa-miR-31 (002,279) or RNU48 (001,006) all from Life Technologies, Germany) and 5 μL RNA (10 ng). RT and qPCR reactions were carried out as described above. All samples were normalized for equal expression of β-actin. For quantification of mRNA expression, the ΔΔ-Ct-method was used.

### Statistical analysis

qPCR data were analysed by means of GraphPad Prism 8 software and were expressed as mean ± SD. For comparisons among the experiments, Welch’s t test and one-way ANOVA with Bonferroni’s test for multiple comparisons were used as appropriate to determine statistical significance. *P* < 0.05 was considered statistically significant.

## Results

### Hierarchical cluster analysis

**Figure 1:**
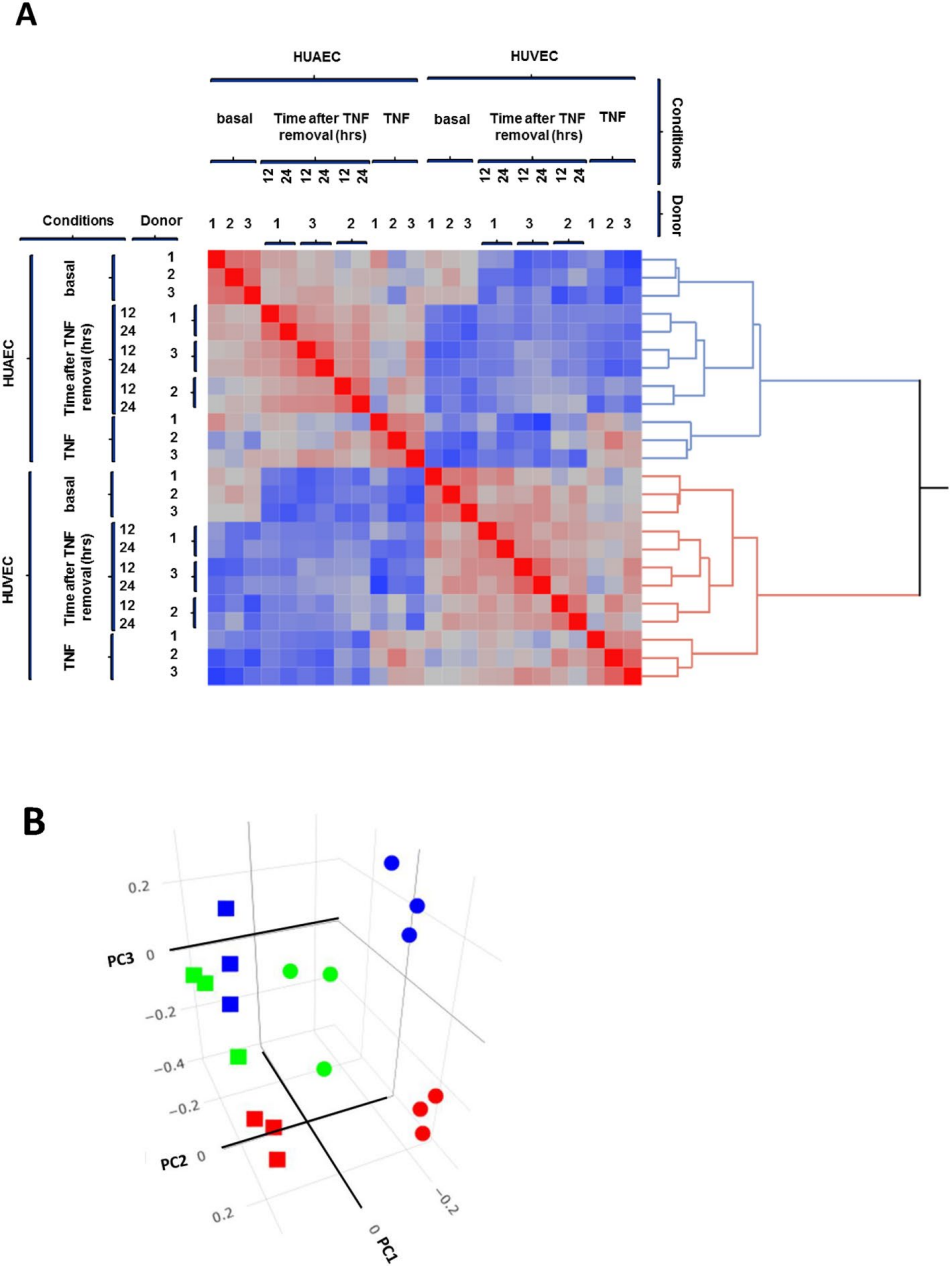
Differential gene expression between HUAEC and HUVEC. (**A**) Hierarchical cluster analysis (HCA) of gene-profiles between HUAEC and HUVEC. On the left- and upper sides are the different conditions (basal, TNF stimulated and TNF removal for 12 or 24 h) as well as the different donors (1–3), on the right side a hierarchical dendogram is shown. (**B**) Principal component analysis of the data-set. Squares represent HUAEC, circles HUVEC, red symbols basal, blue symbols TNF stimulated, green symbols 24 h after TNF removal.

To assess if HUAEC and HUVEC differ in their responsiveness to TNF-α we performed gene-expression profiling on genetically identical HUAEC and HUVEC pairs (n = 3 for each) that were cultured under basal condition or stimulated for 24 h. with TNF-α. We also implemented two additional groups, i.e. cells that were stimulated for 24 h. with TNF-α followed by a culture period of 12 or 24 h. in the absence of TNF-α, allowing the detection of transcriptional differences between HUAEC and HUVEC when returning to homeostasis. Hierarchical clustering analysis (HCA) showed a perfect separation for HUAEC and HUVEC both basal as well as for their treatment (Fig. 1). The largest difference between the gene-profiles was found for the comparisons HUAEC and HUVEC. Within each of these EC subtypes, gene-profiles strongly differed between basal and TNF-α stimulated conditions, while the gene-profiles corresponding to that of TNF-α removal was situated in between these conditions. Of note, gene-profiles of different time points after TNF-α removal in EC of one donor were more similar than between that of similar time points of different donors (Fig. 1). This likely reflects differences in genetic background between the donors and underscores the importance of comparing genetically identical HUAEC and HUVEC pairs.

### Gene-profiles of cultured HUAEC and HUVEC reveal distinct genetic fingerprints

**Figure 2:**
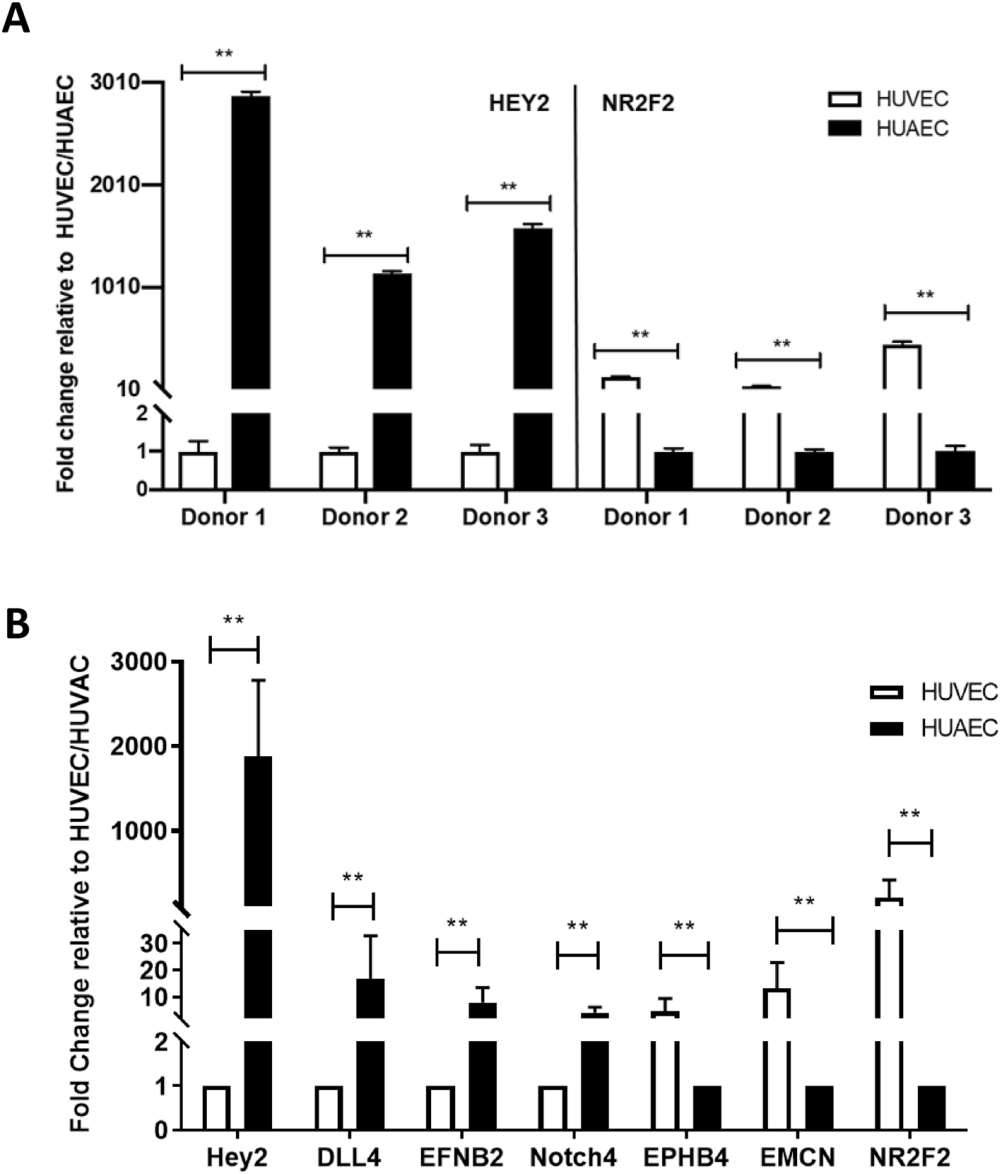
DEG in HUAEC and HUVEC. (**A**) A confirmatory qPCR was performed for HEY2 and NR2F2 for each of the donors. (**B**) The expression of other arterial specific genes (DLL4, EFNB2 and Notch4) and vein specific (EPHB4 and EMCN) were also assessed by qPCR. In (**A**) the result for each donor is expressed as fold change relative to the expression in HUVEC or HUAEC respectively. In (**B**) the results are expressed as mean fold change of the three donors. All qPCR were performed in triplicate. **P < 0.01 (Welch’s t test and one-way ANOVA with Bonferroni’s test for multiple comparisons).

A total of 589 genes were differentially expressed between HUAEC and HUVEC. Of these, 254 genes were significantly stronger expressed in HUVEC (range fold change (Log2): -0.32 to -3.73; range *P*_adj_-values: 0.04 to 2.58E-8), while 335 genes were expressed stronger in HUAEC (range fold change (Log2): 0.34 to 6.82; range *P*_adj_-values: 0.03 to 1.04E-14). The top 10 of differentially expressed genes (DEG) are listed in Table 1. Amongst the DEG two fate determining genes, i.e. HEY2 and NR2F2, important for arterial and venous differentiation^15,16^ were found. In all three different donors, the expression of HEY2 was significantly higher in HUAEC, while the expression of NR2F2 was more pronounced in HUVEC (Fig. 2A). Likewise, the expression of other genes reported to be involved in arteriovenous differentiation were accordingly expressed in HUAEC and HUVEC (Fig. 2B).

**Table 1:** Top 10 of DEG for the comparison HUAEC vs. HUVEC.

Gene	*Lower expressed in HUAEC*		Gene	*Higher expressed in HUAEC*	
	FC (log2)	*P*_*adj*_		FC (log2)	*P*_*adj*_
ADAMTS18	− 4.73	0.0020	HEY2	6.82	1.0E−14
NR2F2	− 4.07	2.5E−08	LOC107986951	6.67	1.9E−08
SESN3	− 4.05	1.9E−05	CPM	5.42	7.1E−08
LOC105379461	− 4.03	0.0001	FAP	4.97	7.9E−08
CDH11	− 3.62	2.5E−08	LAMA2	4.45	2.5E−08
TXNIP	− 3.56	3.6E−05	LOC102723341	4.11	0.0317
LYVE1	− 3.51	0.0267	SLC46A3	4.03	5.2E−05
CXCL11	− 3.51	0.0078	RADGRF2	3.96	1.9E−08
ZNF462	− 3.31	0.0078	TOM1L1	3.93	0.0002
PLAC8	− 3.18	0.0019	SLIT2	3.67	2.5E−08

We next performed Gene-Set Enrichment Analysis (GSEA) on the whole data set to assess if there was enrichment for specific pathways in HUAEC and HUVEC. We could identify a total of 49 pathways of which 5 were upregulated and 44 downregulated in HUAEC relative to HUVEC. The top 10 list of significantly enriched KEGG pathways is shown in Table 2.

**Table 2:** Top 10 of significantly enriched KEGG-Pathways in HUAEC and HUVEC.

KEGG Pathway	NES^a^	P_adj_
hsa05412_Arrhythmogenic_right_ventricular_cardiomyopathy_(ARVC)	1.81	0.0197
hsa04330_Notch_signaling_pathway	1.79	0.0267
hsa05414_Dilated_cardiomyopathy_(DCM)	1.77	0.0187
hsa05410_Hypertrophic_cardiomyopathy_(HCM)	1.64	0.0408
hsa05206_MicroRNAs_in_cancer	1.43	0.0383
hsa05169_Epstein-Barr_virus_infection	− 2.00	0.0041
hsa05034_Alcoholism	− 2.03	0.0041
hsa03430_Mismatch_repair	− 2.13	0.0041
hsa05322_Systemic_lupus_erythematosus	− 215	0.0041
hsa03030_DNA_replication	− 2.51	0.0041

^a^Normalized enrichment score. Positive and negative values reflect upregulation respectively downregulation in HUAEC relative to HUVEC.

### Differences in transcriptional regulation of inflammatory genes

**Figure 3:**
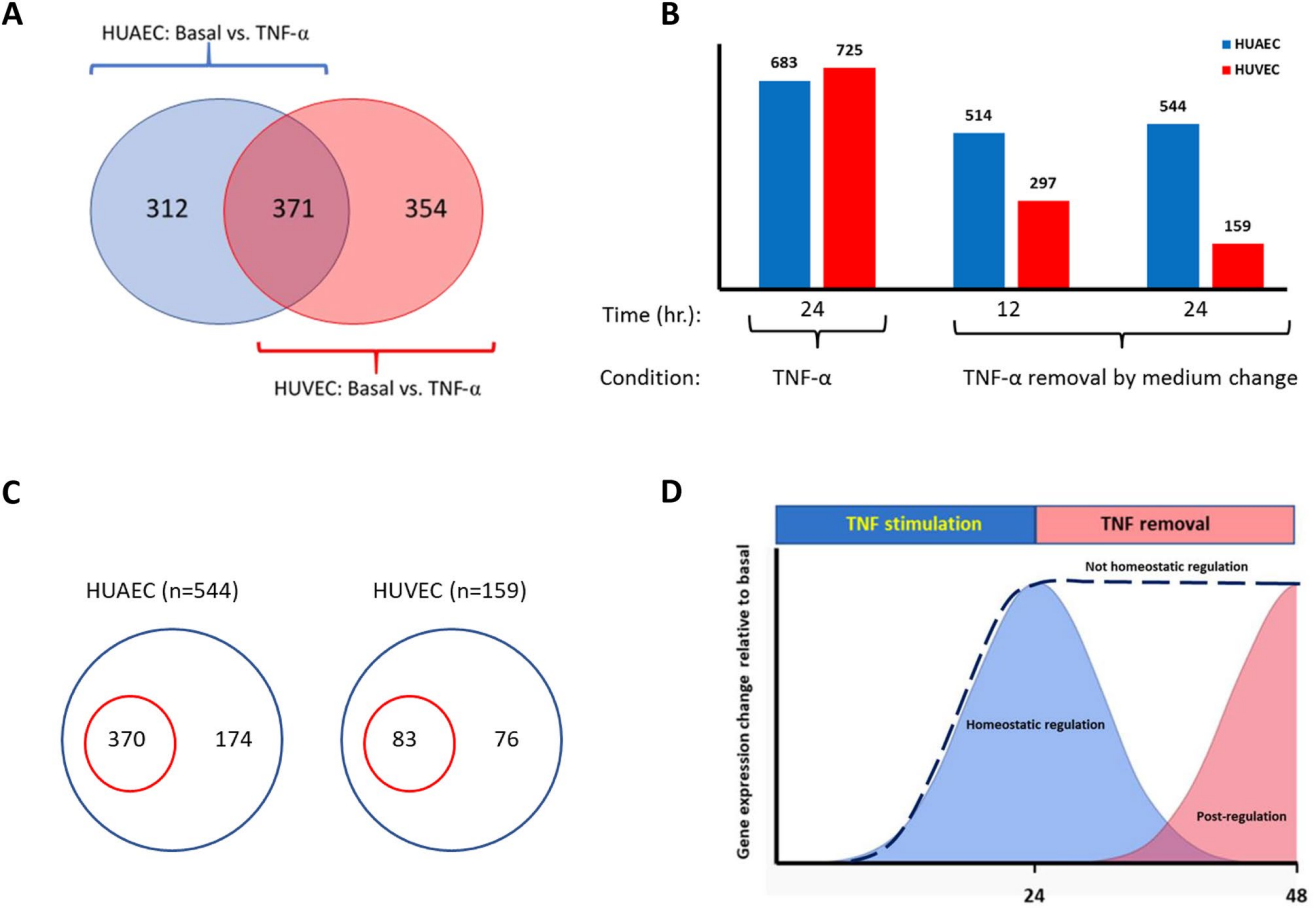
Influence of TNF-α on the gene expression profiles of HUAEC and HUVEC. (**A**) Venn diagram depicting the number of TNF-α regulated genes in HUAEC (blue) and HUVEC (red). (**B**) Quantitative assessment of genes that were regulated by 24 h. of TNF-α stimulation, and after removal of TNF-α by medium change and continued culture for 12 and 24 h. post medium change. (**C**) Venn diagram depicting the number of post-regulated (red circle) genes in HUAEC and HUVEC respectively. The blue circles represent the total number of significantly regulated genes after 24 h. of TNF-α removal (n = 544 in HUAEC; n = 159 in HUVEC). The number in the blue circle represents the numbers of genes that were already significantly regulated by TNF-α before medium change (not homeostatic regulation). (**D**) Types of regulation of gene expression observed. Genes that were significantly regulated after 24 h of TNF-α stimulation but not anymore when TNF-α was removed (homeostatic regulation, blue curve), genes that maintained significantly regulated after TNF-α removal (Not homeostatic regulation, dotted line) and genes that were only significantly regulated when TNF-α was removed (post-regulation, red curve).

The influence of TNF-α on the quantity of regulated genes was not largely different between HUAEC and HUVEC, with a total of 683 and 725 regulated genes respectively. Under TNF-α stimulation 371 were regulated in both HUAEC and HUVEC, whereas 312 and 354 genes were regulated only in HUAEC or HUVEC respectively (Fig. 3A). The top 10 of regulated genes in HUAEC, HUVEC or in both are listed in Tables 3 and 4. A large proportion of top 10 genes that were exclusively down-regulated in HUAEC were classified as long non-coding RNAs (LncRNA and LINC01358), Anti-Sense-RNA (LURAP1L-AS1) or micro RNAs (Mir), (MIRLET7A2 und Mir100). Genes encoding typical pro-inflammatory mediators such as adhesion molecules (E-selectin, VCAM-1, ICAM-1) and chemokines (CxCL3, CxCL5, CxCL8, CxCL10, CxCL11, CCL2) were mostly up-regulated in both HUAEC and HUVEC (Table 4). Confirmatory qPCR showed no significant differences in the expression of these chemokines and adhesion molecules between HUAEC und HUVEC (data not shown).

**Table 3:** Top 10 genes that are exclusively down- or up-regulated in HUAEC or HUVEC upon TNF-α stimulation.

*Top 10 regulated genes in HUAEC*					
Gene	Down-regulated		Gene	Up-regulated	
	FC (log2)	*P*_*adj*_		FC (log2)	*P*_*adj*_
PHGDH	− 2.82	0.006	MMP10	4.91	0.0024
LOC105376603	− 2.74	6.7E−5	HERC6	3.24	0.0413
MIRLET7A2	− 2.41	0.001	TXNIP	3.12	0.0003
ASNS	− 2.30	0.045	TNFSF15	3.03	0.0058
AK5	− 2.15	0.0001	NRP2	1.87	7.2E−5
Mir100	− 2.14	0.0001	PKD1L1	1.86	0.018
SLC7A5	− 2.03	0.007	PDE5A	1.85	0.025
LURAP1L-AS1	− 1.98	0.04	DTX3L	1.85	0.031
ARHGAP20	− 1.88	0.0008	PI3	1.78	0.003
LINC01358	− 1.83	0.02	BDKRB2	1.49	0.010

*Top 10 regulated genes in HUVEC*					
Gene	Down-regulated		Gene	Up-rgulated	
	FC (log2)	*P*_*adj*_		FC (log2)	*P*_*adj*_
LYVE1	− 4.31	0.008	CXCL1	4.87	0.007
PLAC8	− 2.48	0.018	CXCL6	4.23	0.003
APLN	− 2.33	0.0001	LOC1055375913	3.16	0.005
MS4A6A	− 2.32	0.0013	FABP4	2.88	0.001
ABCG2	− 2.08	0.009	PLA1A	2.87	0.0007
NEGR1	− 1.96	0.024	CSF2	2.85	3.02E−7
FBLN5	− 1.91	0.016	RASGEF1B	2.70	0.001
THBD	− 1.88	0.005	LURAP1L	2.61	0.0006
PRICKLE1	− 1.83	0.031	IFI35	2.58	0.022
INPP4B	− 1.75	0.0009	GPR141	2.49	00,002

**Table 4:** Top 10 TNF-α regulated genes in both HUAEC and HUVEC.

*Down-regulated genes*					
Gene	HUAEC		Gene	HUVEC	
	FC (log2)	*P*_*adj*_		FC (log2)	*P*_*adj*_
LOC105377865	− 4.17	8.47E−6	LOC105377865	− 3.40	9.04E−05
MSMP	− 2.74	3.36E−5	MSMP	− 2.43	0.00013
PSAT1	− 2.50	0.013	PSAT1	− 2.37	0.0187
ITGA10	− 2.07	0.0018	PIK3CG	− 1.87	0.0014
SEMA3F	− 1.78	4.79E−7	AXL	− 1.75	0.0024
EDIL3	− 1.72	0.0039	SOX18	− 1.63	0.0033
DPYD	− 1.61	2.02E−5	SEMA3F	− 1.61	1.64E−06
AXL	− 1.59	0.0052	EDIL3	− 1.59	0.0072
EHR1	− 1.55	0.0049	TBC1D2	− 1.44	0.00095
PIK3CG	− 1.53	0.008	EGR1	− 1.43	0.0096

*Up-regulated genes*					
Gene	HUAEC		Gene	HUVEC	
	FC (log2)	*P*_*adj*_		FC (log2)	*P*_*adj*_
SELE	6.22	8.43E−6	SELE	8.56	2.07E−07
CXCL10	6.11	0.0033	CXCL5	7.55	7.90E−08
VCAM1	5.82	8.69E−6	CXCL10	6.96	0.00094
UBD	5.43	1.24E−6	VCAM1	6.81	1.56E−06
TNFRSF9	5.31	4.90E−8	CCL20	6.65	1.86E−06
CXCL5	5.27	4.51E−6	TNFRSF9	6.22	8.31E−09
IFI44L	5.20	0.012	CD69	5.56	4.90E−08
CXCL11	5.13	0.00018	ICAM1	5.38	6.36E−06
CXCL8	4.59	0.023	UBD	5.24	1.83E−06
OAS2	4.33	0.022	CXCL3	5.19	4.44E−07

Upon TNF-α removal the number of regulated genes in HUVEC profoundly decreased in a time dependent manner, while in HUEAC a small decrease was noticed that stabilized between 12 and 24 h (Fig. 3B). The number of genes that were significantly regulated after 24 h. of TNF-α removal consisted of genes that were not—or already significantly regulated directly after TNF-α stimulation (Fig. 3C). Therefore, three types of gene regulation could be identified: genes that were significantly regulated after 24 h. of TNF-α stimulation but not anymore when TNF-α was removed, genes that maintained significantly regulated after TNF-α removal and genes that were only significantly regulated when TNF-α was removed. These types of regulation were tentatively assigned as homeostatic -, not homeostatic- and post-regulation respectively (Fig. 3D). HUAEC and HUVEC quantitatively differed in these types of gene regulation, with relatively less genes returning to homeostasis and relative more genes being post-regulated in HUAEC (Table 5). Confirmatory qPCR of selected genes for the different types of regulation is shown in supplementary Table 1.

**Table 5:** Differences in types of gene regulation between HUAEC and HUVEC.

Type of gene regulation^a^	HUAEC	HUVEC	*P-value*^b^
Homeostatic regulation	509 (74.5%)^C^	649 (89.5%)	< .0001
Not homeostatic regulation	174 (25.5%)	76 (10.5%)	< .0001
Post-regulation	370 (68%)^d^	83 (52.2%)	0.003
Not homeostatic regulation	174 (32%)	76 (47.8%)	0.003

^a^For homeostatic vs. not homeostatic regulation the number of genes calculated from the difference between the number of significantly regulated genes 24 h after TNF-α stimulation and the number of genes that remained significant after TNF-α removal.

^b^Statistics was performed by Chi-square test.

^c^The percentage between brackets are relative to the number of significantly regulated genes after 24 h of TNF-α stimulation.

^d^The percentage between brackets are relative to the number of significantly regulated genes after 24 h of TNF-α removal.

Genes that belong to homeostatic regulation were further analysed in the Gene-ontology and KEGG database. For HUAEC gene enrichment was found in 30 pathways (10 GOTERM and 20 KEGG pathways) and for HUVEC 70 pathways (39 GOTERM and 31 KEGG pathways) were enriched. With the exception of one, i.e. GOTERM GO:0070498 ~ interleukin-1-mediated signalling pathway, all pathways in which enrichment was found for HUAEC were also found for HUVEC. Hence, for homeostatic regulation our analysis disclosed 41 Pathways that were exclusively enriched for HUVEC. The Top 10 list of GO terms or KEGG pathways is shown in supplementary Table 2. Genes that were post-regulated were subjected to overrepresentation analysis (ORA) for GO terms and KEGG pathways. For the 83 post-regulated genes in HUVEC no significant enrichment for GO terms or KEGG pathways was found. In contrast 370 post-regulated genes in HUAEC a significant up-regulation was found for 18 KEGG pathways and down-regulation for 92 GO terms and 7 KEGG pathways (supplementary Fig. 1).

### MicroRNAs (mir) expression in HUAEC and HUVEC

MicroRNAs can modulate inflammatory processes through their regulatory effects on gene expression by degrading complementary mRNA targets and inhibiting translation^17^. Amongst the genes that were down-regulated exclusively in HUAEC by TNF-α, mir, or long non-coding RNAs (LncRNA) were found in the top 10 list of most down-regulated genes. We therefore assessed if mir expression per se differ between HUAEC and HUVEC (Table 6). Under basal conditions, a total of 13 mir were more expressed in HUAEC. Upon TNF-α stimulation 7 out of 13 were downregulated, of which mir100 was the most affected (FC (Log2): -2,14; P_adj_: 3,2E-5) (Table 6). Because mir100 has been reported to suppress inflammation^18–21^ we looked for validated mir100 target in the miRWalk data base^22^ and assessed if these targets were influenced by TNF-α in the arrays. A total of 17 target mRNAs were significantly modulated by TNF-α, of which 7 were upregulated (Table 7). TNF-α mediated down-regulation of mir100 and upregulation of the mir100 target PDE5A was confirmed by qPCR (supplementary Table 1).

**Table 6:** Mir expression in HUAEC and HUVEC: Influence of TNF-α.

Gene	HUAEC vs. HUVEC		TNF-α vs. Basal in HUAEC	
	FC (log2)	*P*_*adj*_	FC (log2)	*P*_*adj*_
Mir 21	1.82	0.0007		ns
Mir100	1.56	0.0006	− 2.14	3.2E−5
MIRLET7A2	1.50	0.032	− 2.41	0.001
MIR31	1.50	0.0085	− 1.40	0.017
LURAP1L-AS1	1.46	ns	− 1.98	0.04
MIR634	1.43	0.043		ns
MIR222	1.36	0.0009	− 1.61	0.019
MIR218-1	1.22	0.009		ns
MIR503	1.03	0.009	− 1.15	0.027
MIR137HG	0,98	0.009		ns
MIR181B1	0.98	0.043		ns
MIR32	0.96	0.034		ns

**Table 7:** mir100 targets that are significantly influenced by TNF-α in HUAEC and HUVEC.

Gene	HUAEC		HUVEC	
	FC (log2)	*P*_*adj*_	FC (log2)	*P*_*adj*_
**PDE5A**	1.86	0.02528	1.43	ns
**RCSD1**	1.45	0.00951	0.97	ns
**YAE1D1**	1.02	0.00588	0.69	ns
**S1PR2**	0.85	0.00146	0.40	ns
**IRGQ**	0.63	0.04731	0.29	ns
**RNF207**	0.59	0.01832	0.34	ns
**STX6**	0.58	0.01935	0.43	ns
PXK	− 0.53	0.02503	− 0.23	ns
THUMPD2	− 0.56	0.02641	− 0.21	ns
SHANK1	− 0.76	0.04942	− 0.05	ns
CELF2	− 0.78	0.00688	− 0.14	ns
LSM11	− 0.81	0.00355	− 0.48	ns
MAP4K4	− 0.87	0.00087	− 0.34	ns
ODC1	− 0.88	0.02237	− 0.61	ns
LAMA5	− 0.91	0.01165	− 0.48	ns
ST3GAL5	− 1.25	0.03425	− 0.58	ns
FRMD5	− 1.50	0.03123	− 0.44	ns

## Discussion

The present study underlies the hypothesis that arterial and venous endothelial cells differ in their ability to support inflammation and in their ability to return to homeostasis after an inflammatory stimulus. The hypothesis was tested by assessing how gene-expression profiles were changed in arterial and venous endothelial cells by TNF-α and when homeostasis was reinstated. We used genetically identical HUAEC and HUVEC to exclude that genetic polymorphisms were underlying differences between these cell types. The main findings of our study are the following. Firstly, TNF-α regulates the expression of different sets of transcripts that are significantly changed only in HUAEC, only in HUVEC or changed in both. Secondly, three types of gene regulation were identified, i.e. genes that were significantly regulated after 24 h. of TNF-α stimulation but not anymore when TNF-α was removed (homeostatic regulation), genes that maintained significantly regulated after TNF-α removal (not homeostatic regulation) and genes that were only significantly regulated when TNF-α was removed (post-regulation). Thirdly, HUAEC and HUVEC quantitatively differed in these types of gene regulation, with HUAEC displaying less homeostatic – and more post-regulation.

Genome wide gene-expression profiling has been used in a number of studies to demonstrate heterogeneity amongst different EC subtypes, including arterial-, venous-, microvascular -, macrovascular—and lymphatic EC^23–26^. Although some of these studies reported that the cell culture process compromises specific expression profiles, in our study HCA was able to distinguish HUAEC from HUVEC. Differences in gene expression profiles between HUAEC and HUVEC were roughly in line with that reported for arterial and venous EC^23^. Since gene-expression profiles of freshly isolated HUAEC and HUVEC were not assessed, it cannot be excluded that some phenotypic drift did occur as a consequence of cell isolation and culturing. In the study of Aranguren et al^24^ arterial EC could only be distinguished from venous EC when freshly isolated EC were used. In contrast to their study we used umbilical cords as only source for EC, which may contribute to a more homogeneous data set and therefore to a better dissection of both cell types.

As compared to HUVEC, GSEA revealed upregulation of 5 and downregulation of 44 pathways in HUAEC. As expected for arterial endothelial cells^27,28^ the Notch signaling pathway was upregulated, with Hey2, DLL4, Hey1 and Notch4 being the most affected genes in this pathway (supplementary Table 3). Three other pathways that were related to cardiomyopathies and one pathway that was defined as MicroRNAs_in_cancer (hsa05206) were also upregulated in HUAEC. In the latter pathway CD44, mir21 and mir100 were the most prominently upregulated genes (supplementary Table 4). While CD44 expression was not affected by TNF-α in neither of the studied EC types, in HUAEC the anti-inflammatory mir100^18^ was significantly downregulated by TNF-α which was still noticed 24 h. after TNF-α removal.

Although our data demonstrate that TNF-α to some extent differentially affects gene expression profiles in HUAEC and HUVEC, the most striking difference between HUAEC and HUVEC was the spatiotemporal regulation of genes upon TNF-α stimulation and removal. While in HUVEC homeostatic regulation, i.e. change in gene-expression returning to baseline upon removal of TNF-α, was more common, in HUAEC post-regulation, i.e. gene-expression only changed after TNF-α removal, was more frequently observed. Homeostatic regulated genes in HUAEC and HUVEC were mostly typical pro-inflammatory mediators, e.g. adhesion molecules and cyto-/chemokines. Post-regulated genes in HUAEC were more diverse including junctional protein, solute carriers and signaling molecules. Although we did not study if the expression level of post-regulated genes in our experimental setting is different from that in quiescent endothelial cells, several of the post-regulated genes including PDGFD (3,2 times higher compared to cultured HUAEC) are reported to be undetectable in the healthy vasculature. Importantly, increased expression of PDGFD is found in many vascular and cardiovascular diseases^29,30^.

Limitations of the study: Even though our study has revealed differences between arterial and venous EC in the context of inflammation and resolution that might be relevant for chronic inflammatory diseases, our study is in essence explorative and does not disclose underlying mechanisms that explain these differences. Moreover, the use of HUAEC and HUVEC may not be the most representative endothelial cells to assess differences in transcriptional regulation between arterial and venous endothelial cell. Hence, generalization of the current findings should be taken with care. TNF-α is crucially involved in the pathogenesis and progression of a number of inflammatory conditions, yet many other factors e.g. lipid and pro-resolving mediators will influence the course of inflammation and may differentially influence gene-expression in arterial and venous EC. Moreover, since the cultured endothelial cells were removed from their microenvironments, a phenotypic drift to some extent might occur, which otherwise would not be present in venous and arterial endothelial cells. We acknowledge that these important limitations warrant further optimization of the experimental set-up, e.g. the inclusion of sheer stress, leucocytes or leucocyte derived factors in future experiments. However, our data do suggest that upon TNF-α stimulation changes in gene-expression seem to be more persistent in HUAEC as compared to HUVEC. Whether this also holds true for other pro-inflammatory cytokines or for complex models that better mirror in vivo organ inflammation remain to be assessed. It should also be mentioned that in contrast to adult vasculature the umbilical artery carries non-oxygenated blood while the umbilical vein has the oxygenated blood. The in vitro cultured HUAEC and HUVEC were exposed to the same oxygen pressure. It therefore would be prudent to be cautious in concluding that in the adult vasculature arterial and venous endothelial cells respond differently towards TNF-α.

### Supplementary Information

Supplementary Information 1.

Supplementary Information 2.

Supplementary Information 3.

Supplementary Information 4.

## Data Availability

The raw and normalized data are deposited in the Gene Expression Omnibus database (http://www.ncbi.nlm.nih.gov/geo/; accession No. GSE179478).
