# Supplementary figures and images for: Gene regulation for inflammation and inflammation resolution differs between umbilical arterial and venous endothelial cells
Source: Sci Rep. 2023 Sep 27;13:16159. doi: 10.1038/s41598-023-43142-6 (PMC10533526; doi:10.1038/s41598-023-43142-6)

## Slide 1
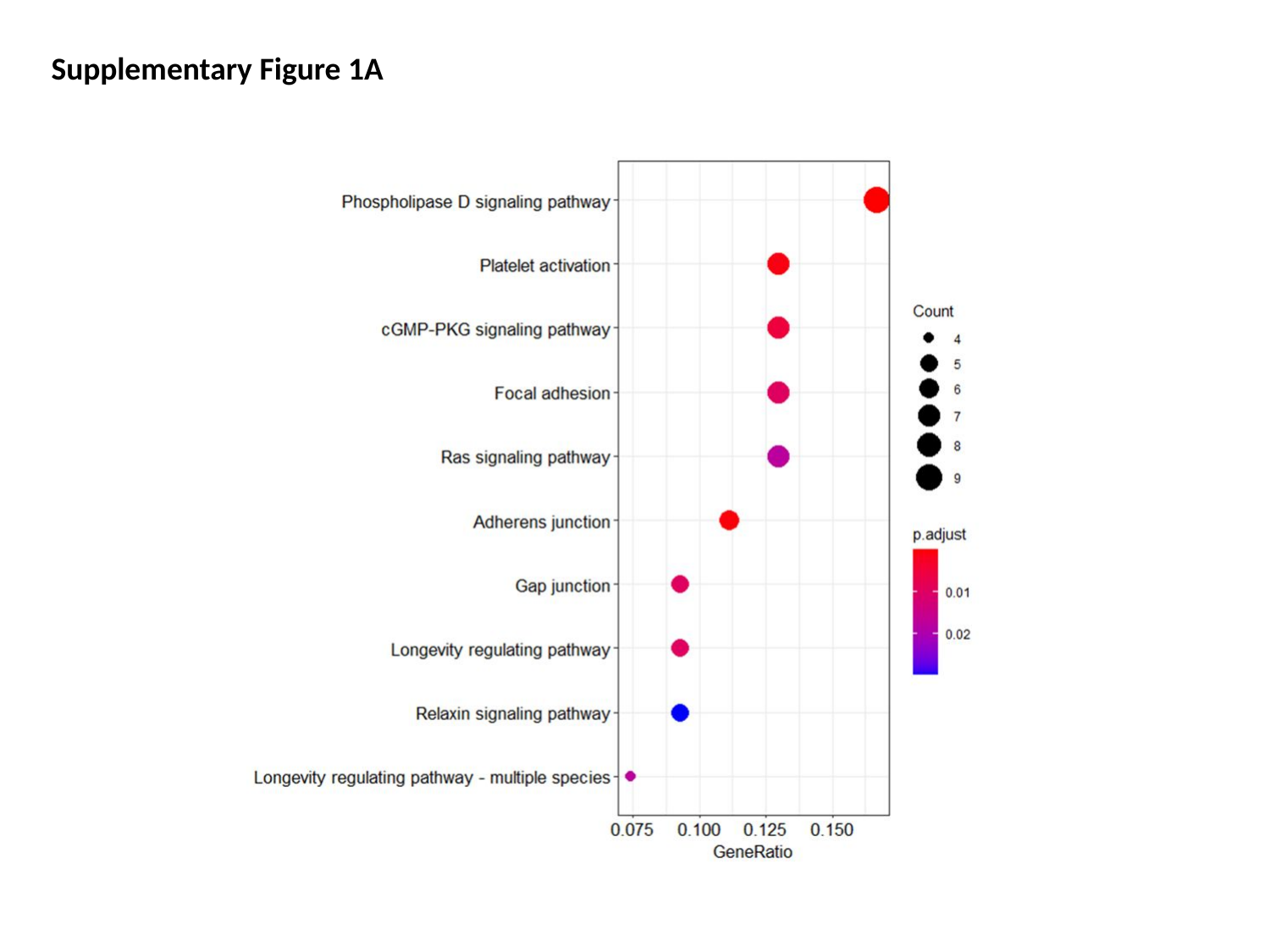

Supplementary Figure 1A

Supplement: Supplementary file 1 — Supplementary Information 1. [file 41598_2023_43142_MOESM1_ESM.pptx]

## Slide 1
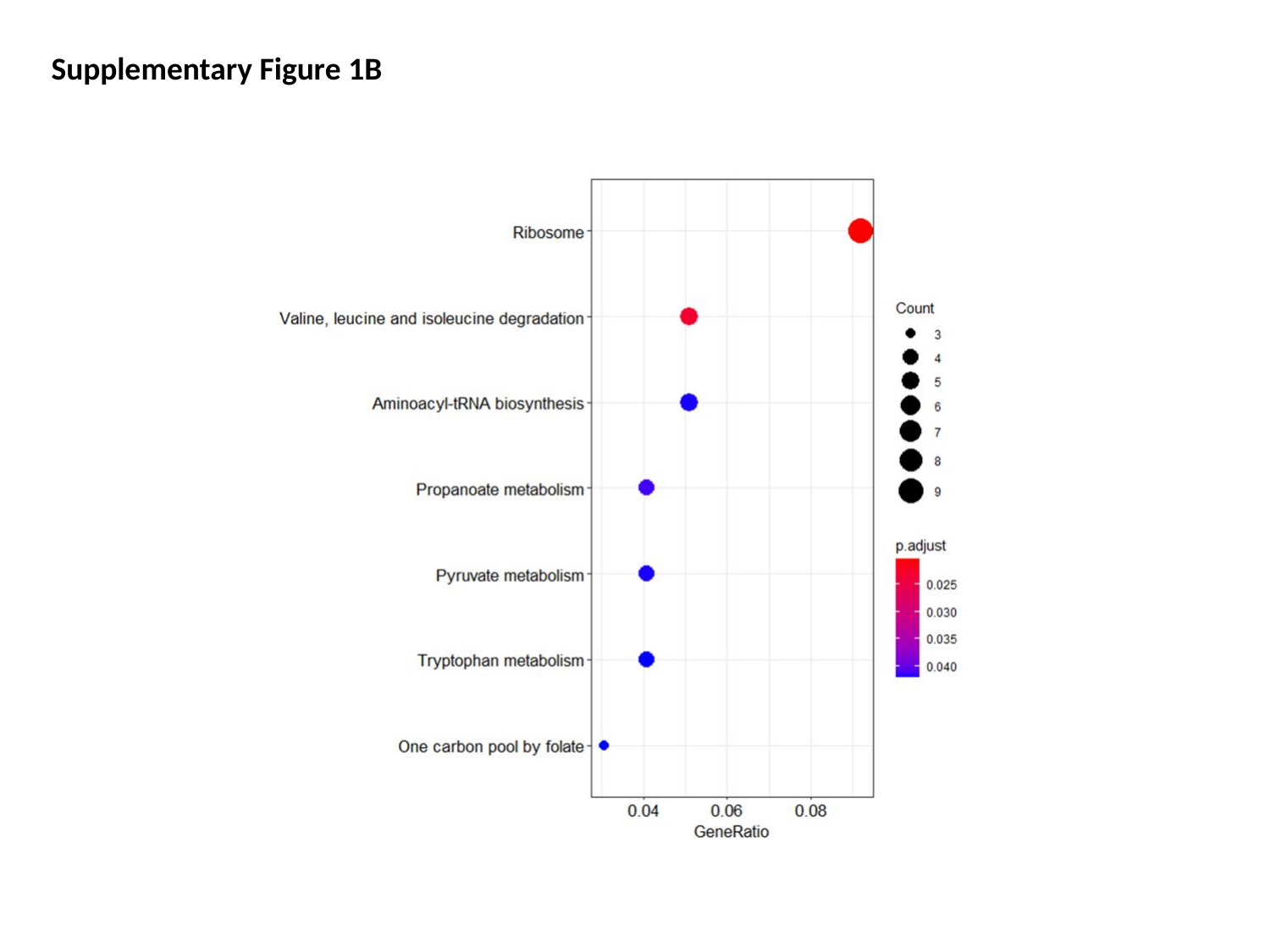

Supplementary Figure 1B

Supplement: Supplementary file 2 — Supplementary Information 2. [file 41598_2023_43142_MOESM2_ESM.pptx]

## Slide 1
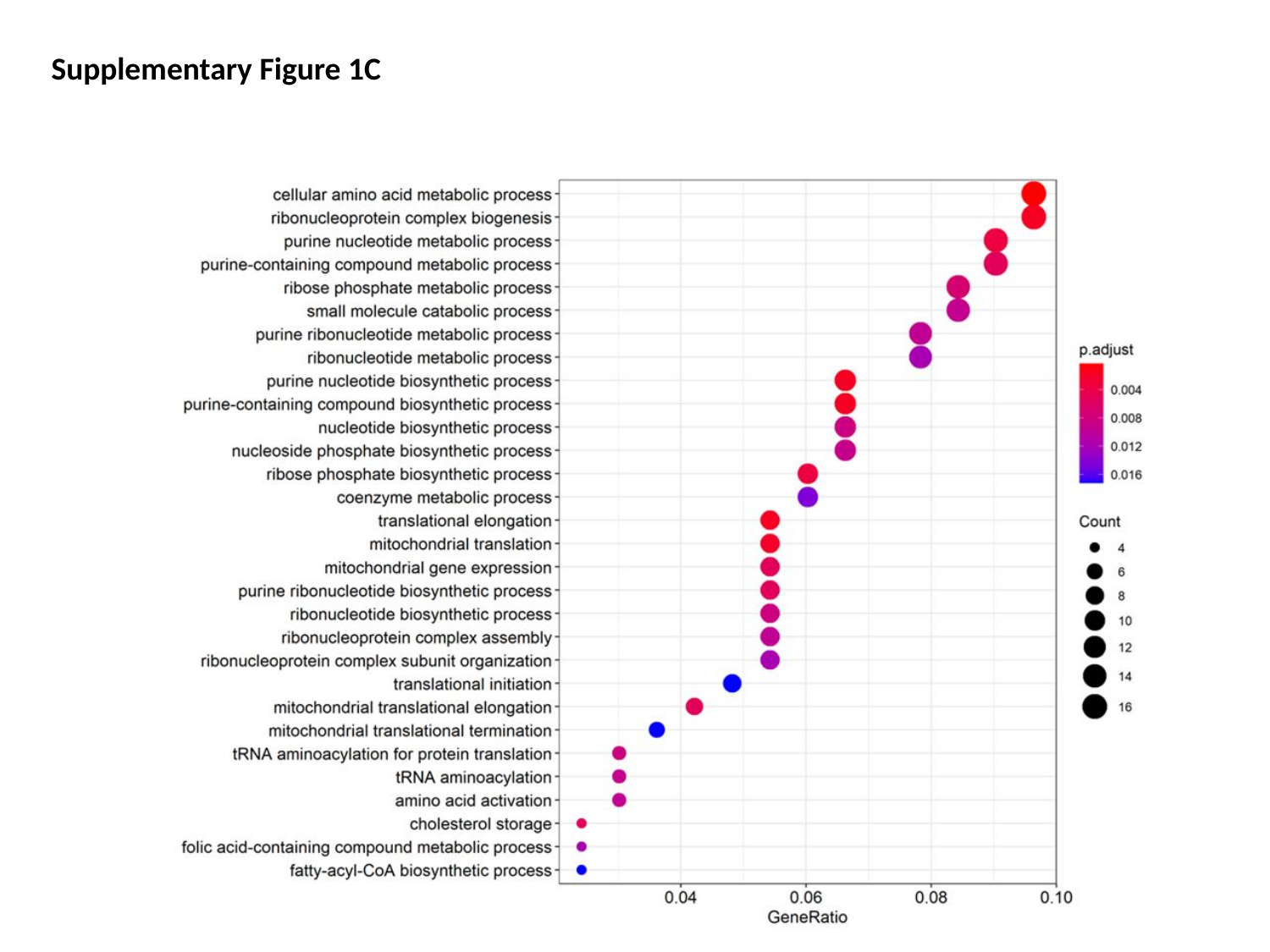

Supplementary Figure 1C

Supplement: Supplementary file 3 — Supplementary Information 3. [file 41598_2023_43142_MOESM3_ESM.pptx]
