# Supplementary material for: Gene regulation for inflammation and inflammation resolution differs between umbilical arterial and venous endothelial cells
Source: Sci Rep. 2023 Sep 27;13:16159. doi: 10.1038/s41598-023-43142-6 (PMC10533526; doi:10.1038/s41598-023-43142-6)
Supplement: Supplementary file 4 — Supplementary Information 4. [file 41598_2023_43142_MOESM4_ESM.docx]

**Supplementary figure 1:** Dot plot analysis of the most significantly enriched GO terms or KEGG pathways for post-regulated genes in HUAEC. In **A** and **B** KEGG pathways that were up-regulated (**A**) or down-regulated (**B**), in **C** GO terms that were up-regulated.


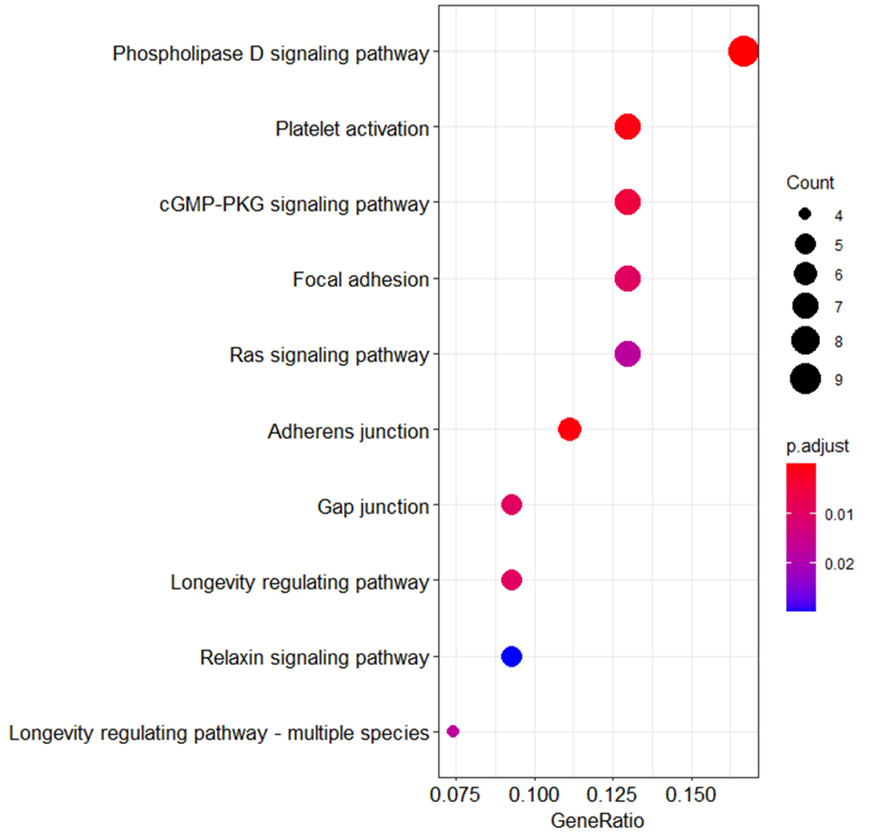
**Figure 1**

**A**

**B**

**
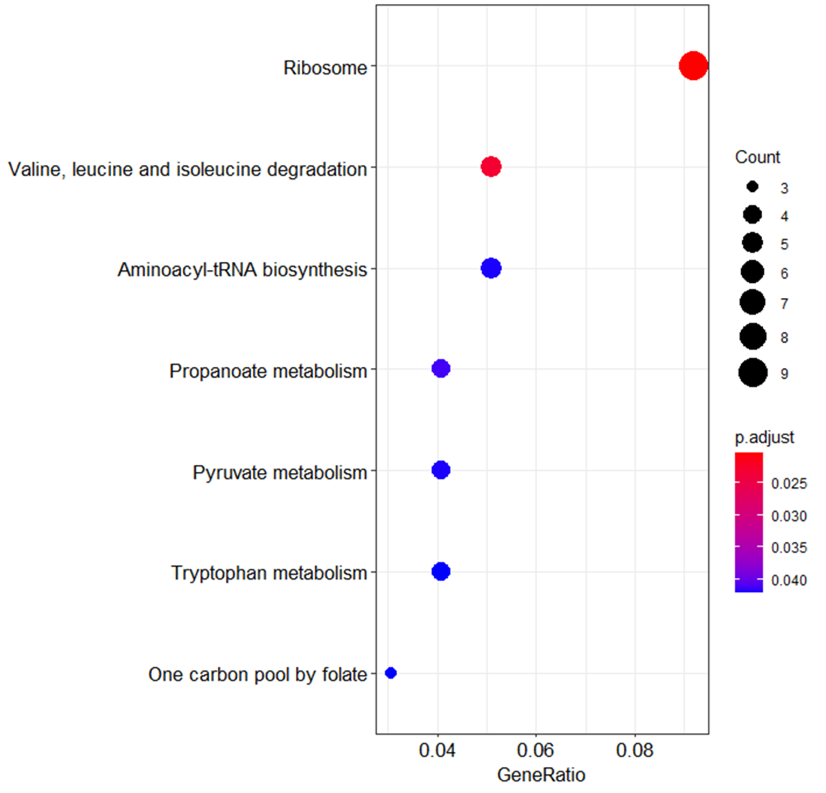
**

**
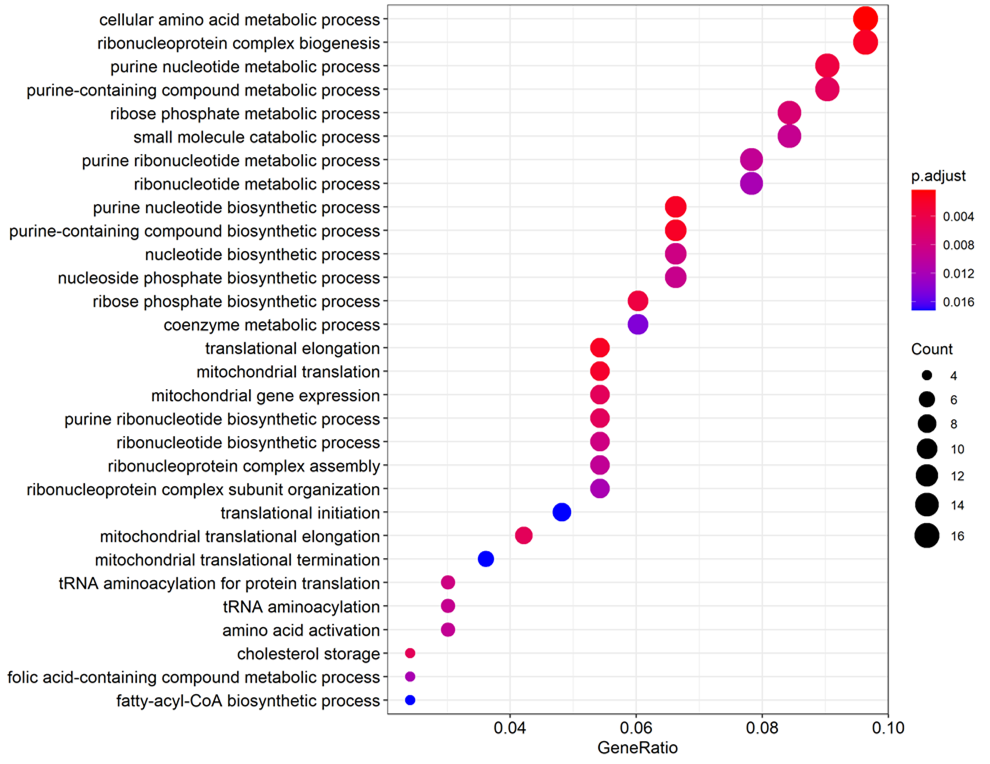
C**

**Supplementary table 1:** Top 10 list of GO terms or KEGG pathways for homeostatic regulated gene that were exclusively enriched in TNF-α simulated HUVEC

| **Category** | **Term** | **FDR** |
| --- | --- | --- |
| GOTERM | GO:0006955~immune response | 9.83E-05 |
| GOTERM | GO:0060326~cell chemotaxis | 4.80E-04 |
| GOTERM | GO:0007165~signal transduction | 4.80E-04 |
| KEGG | hsa05134:Legionellosis | 6.94E-04 |
| GOTERM | GO:0051607~defense response to virus | 7.45E-04 |
| GOTERM | GO:0070098~chemokine-mediated signaling pathway | 8.60E-04 |
| GOTERM | GO:0050727~regulation of inflammatory response | 0.0013 |
| GOTERM | GO:0033209~tumor necrosis factor-mediated signaling pathway | 0.0015 |
| GOTERM | GO:0051092~positive regulation of NF-kappaB transcription factor activity | 0.0015 |
| GOTERM | GO:0006935~chemotaxis | 0.0019 |

**Supplementary table 2:** confirmatory qPCR for gene expression and type of regulation

| **HUAEC** | | **Med. vs. TNF** | | **Med. vs. TNF wash-out** | |
| --- | --- | --- | --- | --- | --- |
| **Gene** | **Reg. Type^a^** | **FC** | **P-value** | **FC** | **P-value** |
| CLC4A4 | P | 0.3 ± 0.4 | ns^b^ | 2.1 ± 0.2 | 0.03 |
| CXCL3 | H | 66.6 ± 1.0 | 0.01 | 1.0 ± 0.5 | ns |
| PDE5A | H | 3.7 ± 0.1 | 0.02 | 1.4 ± 0.6 | ns |
| NPR3 | P | 0.3 ± 0.1 | ns | 2.8 ± 0.1 | 0.02 |
| CXCL5 | H | 43.9 ± 1.7 | 0.01 | 1.1 ± 0.4 | ns |
| TXNIP | NH | 2.8 ± 0.4 | 0.01 | 3.3 ± 0.02 | 0.01 |
| B2M | NH | 8.4 ± 1.4 | 0.01 | 5.6 ± 0.7 | 0.01 |
| Mir100 | NH | 0.72 ± 0.05 | 0.01 | 0.54 ± 0.01 | 0.01 |

**^a^**: type of regulation (Reg.Type) (Homeostatic (H), not-homeostatic (NH), post-regulation (P)), **^b^**: not significant (ns)

**Supplementary table 3**: Significant changes in the Notch signalling pathway (hsa04330) in HUAEC

| **Gene** | **FC (log2)** | ***P_adj_*** |
| --- | --- | --- |
| HEY2 | 6.82 | 2.1E-15 |
| DLL4 | 2.86 | 2.03E-05 |
| HEY1 | 2.72 | 0.0021 |
| NOTCH4 | 1.24 | 0.0008 |

**Supplementary table 4:** Significant changes in the MicroRNAs in cancer (hsa05206) pathway in HUAEC

| **Gene** | **FC (log2)** | ***P_adj_*** |
| --- | --- | --- |
| CD44 | 3.28 | 0.002421 |
| MIR21 | 1.82 | 0.000498 |
| MIR100 | 1.56 | 0.000469 |
| EFNA5 | 1.51 | 0.001186 |
| NOTCH4 | 1.24 | 0.000881 |
| TPM1 | 1.21 | 0.003756 |
| MET | -0.84 | 0.001342 |
